# Within-host acquisition of colistin-resistance of an NDM-producing *Klebsiella quasipneumoniae* subsp. *similipneumoniae* strain through the insertion sequence-*903B*-mediated inactivation of *mgrB* gene in a lung transplant child in China

**DOI:** 10.3389/fcimb.2023.1153387

**Published:** 2023-08-31

**Authors:** Yongli Wu, Jiankang Zhao, Ziyao Li, Xinmeng Liu, Yanning Hu, Feilong Zhang, Yulin Zhang, Danni Pu, Chen Li, Xianxia Zhuo, Huihui Shi, Binghuai Lu

**Affiliations:** ^1^ Peking Union Medical College, Chinese Academy of Medical Sciences, Beijing, China; ^2^ Laboratory of Clinical Microbiology and Infectious Diseases, Department of Pulmonary and Critical Care Medicine, National Center for Respiratory Medicine, China-Japan Friendship Hospital, Beijing, China; ^3^ Institute of Respiratory Medicine, Chinese Academy of Medical Sciences, Beijing, China; ^4^ Institute of Clinical Medical Sciences, China-Japan Friendship Hospital, Beijing, China; ^5^ Department of Pulmonary and Critical Care Medicine, Peking University China-Japan Friendship School of Clinical Medicine, Beijing, China; ^6^ Department of Pulmonary and Critical Care Medicine, Capital Medical University China-Japan Friendship School of Clinical Medicine, Beijing, China; ^7^ Department of Clinical Laboratory, Affiliated Nantong Hospital of Shanghai University, Nantong, China

**Keywords:** *Klebsiella quasipneumoniae* subsp. *similipneumoniae*, colistin, *mgrB* gene, insertional sequence, within-host evolution

## Abstract

**Background:**

Colistin, as the antibiotic of “last resort” for carbapenem-resistant *Klebsiella*, develop resistance during administration of this antimicrobial agent. We identified an NDM-1-producing *Klebsiella quasipneumonuae* subsp. *similipneumoniae* (KQSS) strain KQ20605 recovered from a child, which developed resistance to colistin (KQ20786) through acquiring an IS*903B* element between the -27^th^ and -26^th^ bp of *mgrB* promoter region after 6-day colistin usage.

**Objectives:**

The aim of this study is to explore the source of IS*903B* in the disruptive *mgrB* gene and its underlying mechanisms.

**Materials and methods:**

Antibiotics susceptibility testing was conducted *via* microbroth dilution method. The *in vitro* colistin-induced experiment of KQ20605 was performed to mimic the *in vivo* transition from colistin-sensitive to resistant. Whole-genome sequencing was used to molecular identification of colistin resistance mechanism.

**Results:**

The IS*903B* element integrated into *mgrB* gene of KQ20786 had a 100% nucleotide identity and coverage match with one IS*903B* on plasmid IncR, and only 95.1% (1005/1057) identity to those on chromosome. *In vitro*, upon the pressure of colistin, KQ20605 could also switch its phenotype from colistin-sensitive to resistant with IS elements (e.g., IS*903B* and IS*26*) frequently inserted into *mgrB* gene at “hotspots”, with the insertion site of IS*903B* nearly identical to that of KQ20786. Furthermore, IS*26* elements in this isolate were only encoded by plasmids, including IncR and conjugative plasmid IncN harboring *bla*_NDM_.

**Conclusion:**

Mobilizable IS elements on plasmids tend to be activated and integrated into *mgrB* gene at “hotspots” in this KQSS, thereby causing the colistin resistance emergence and further dissemination.

## Introduction

Carbapenemase-producing *Klebsiella* spp. are key pathogens for global nosocomial dominance (Tzouvelekis et al., 2012). Colistin is currently considered the “last resort” antibiotic for carbapenemase-producing *Klebsiella* spp. Yet, over the years, colistin-resistant *Klebsiella* strains have been emerging (Biswas et al., 2012; Grundmann et al., 2017; Binsker et al., 2022), further increasing the threat to public health. The underlying mechanism of this shift remains unclear, thus posing a major concern.

*Klebsiella* mainly consisted of three phylogroups, *K. pneumoniae* (phylogroup I), *K. quasipneumoniae* (phylogroup II), and *K. variicola* (phylogroup III). *K. quasipneumoniae*, subclassified into *K. quasipneumoniae subsp. similipneumoniae* (phylogroup IIB, hereafter referred to as KQSS), and *K. quasipneumoniae subsp. quasipneumoniae* (phylogroup IIA), are new species that can be distinguished from *K. pneumoniae* (Long et al., 2017). In a Japanese study that examined 119 *Klebsiella* isolates collected from blood samples, 11 (9.2%) species were identified as *K. quasipneumoniae* using genomic analysis but misidentified as *K. pneumoniae* in routine microbiology (Imai et al., 2019), thus resulting in underreported infections caused by this species (Long et al., 2017). Ahmed et al. suggested that colistin resistance in *Klebsiella* is predominantly mediated by the decrease of negative charge in the outer membrane due to modified lipopolysaccharide (LPS), thereby impairing the binding affinity between colistin and Lipid A (El-Sayed Ahmed et al., 2020). The modifications of LPS include 4-amino-4-deoxy-l-arabinopyranose (Ara4N) and phosphoethanolamine (pEtN) to lipid A via *arnBCADTEF* (*pmrK*) or *pmrC* products, respectively, which are mainly regulated by two-component regulatory systems (TCS) involving *pmrAB*, *phoPQ*, and *crrAB* genes (El-Sayed Ahmed et al., 2020). In addition, Lippa et al. found that the product of *mgrB* has a negative feedback effect on *phoPQ*, weakening the modification of LPS (Lippa and Goulian, 2009). Moreover, Liu et al. discovered that acquiring mobile genetic elements carrying a member of the mobilized colistin resistance (*mcr*) gene family may also lead to lipid A modification (Liu et al., 2016).

The insertion sequence (IS), harboring one or two transposase (*tnp*) genes, is a common mobile element that acts through a transposase (Partridge et al., 2018). After being transposed, transposase usually generates direct repeats (DRs) at both ends (Weinert et al., 1983). A previous study found that IS elements with DRs had a lower transposition frequency than those without (Zhang et al., 2021). Today, IS elements-mediated insertional disruption and inactivation of the chromosomal *mgrB* gene and/or its promoter region (hereafter referred to as *mgrB* gene) are considered the main cause of colistin resistance (Cannatelli et al., 2013; Cannatelli et al., 2014; Poirel et al., 2015; Shankar et al., 2019). As previously documented, the types of IS elements inserted in the *mgrB* gene often involve IS*Kpn25*, IS*903B*, IS*Kpn14*, and IS*Kpn26*, etc. (Cannatelli et al., 2014; Olaitan et al., 2014; Poirel et al., 2015; Esposito et al., 2018; Yang et al., 2020; Silva et al., 2021; Yan et al., 2021). It has been reported that 60% of *K. pneumoniae* carry IS*903B* elements on its plasmids (Fordham et al., 2022). The dominant Inc groups harboring IS*903B* elements are fusion plasmids IncHI2A + IncHI2, IncFIB(K), and IncR, 12.5% of which co-carry *IS903B* elements and carbapenemase genes, with *bla*_NDM-1_ dominating (Fordham et al., 2022). The possible association between IS*903B* elements inserted in the *mgrB* gene and colistin resistance has also been documented (Yang et al., 2020). However, little is known about the source of IS*903B* in the disruptive *mgrB* gene and its underlying mechanisms.

Herein, we reported a New Delhi metallo-β-lactamase (NDM-1)-producing KQSS strain KQ20605 isolated from a lung transplant of a 7-year-old boy, who developed resistance to colistin (KQ20786) after a 6-day colistin administration. We further analyzed the possible mechanism of this transition from colistin-sensitive to resistant.

## Materials and methods

### Bacterial isolates

Colistin-resistant KQSS strain KQ20786 and its parental colistin-susceptible strain KQ20605 were collected from bronchoalveolar lavage fluid (BALF) from a 7-year-old child admitted to China-Japan Friendship Hospital (CJFH), Beijing, China on March 2019. These two strains were not obtained at admission or within 48 hours of admission to the hospital. The KQ20605 was recovered on day 9 after admission, while KQ20786 was isolated on day 15. Identification of species was performed using matrix-assisted laser desorption–ionization time-of-flight mass spectrometry (MALDI-TOF MS, Bruker Daltonik, Bremen, Germany). Clinical data on the case were retrospectively reviewed.

### Antibiotics susceptibility testing

Susceptibility to ticarcillin-clavulanic acid, piperacillin-tazobactam, ceftazidime, cefoperazone-sulbactam, cefepime, aztreonam, imipenem, meropenem, amikacin, tobramycin, ciprofloxacin, doxycycline, and minocycline was measured using VITEK^®^2 system (BioMérieux, Marcy l’Étoile, France) following the manufacturer’s instructions. The minimum inhibitory concentrations (MICs) of colistin and tigecycline were determined by broth microdilution as recommended by the Clinical and Laboratory Standards Institute (CLSI, 2022). Interpretation of antibiotic susceptibility was also in line with CLSI, 2022 criteria (CLSI, 2022), except for tigecycline, which followed the standard of USA Food and Drug Administration (FDA) breakpoints (https://www.fda.gov/drugs/development-resources/tigecycline-injection-products). *Escherichia coli* ATCC 25922 was used as a control.

### Population analysis profile

PAP was conducted on the colistin-susceptible KQ20605 isolate to exclude the possibility of colistin-heteroresistance, as previously described (Mashaly and Mashaly, 2021). Briefly, serial 100-fold dilutions ranging from 10^8^ to 10^2^ CFU/ml were prepared from overnight culture. Then, 10μL of each dilution was spread on Mueller-Hinton agar (MHA) plates containing 0, 0.5, 1, 2, 3, 4, 5, 6 and 8 mg/L of colistin, respectively. Next, the plates were incubated for 24 h at 37°C. Isolates that grew on plates with colistin concentrations >2mg/L were considered heteroresistant (Li et al., 2006).

### Complementation experiments

Complementation experiments were conducted to clarify the association between *mgrB* insertion and colistin resistance. Briefly, the apramycin-resistant plasmid pDK6-*mgrB* was used. A pDK6 derivative carrying a cloned copy of wild-type *mgrB* gene and some flanks were amplified by PCR using primers *mgrB*-F/R (F: AGCCAAGCTTGCATGCCTGCAGAGCCAGCGATGCCAGATTTA, R: ACAGGAAACAGAATTCGAGCTCCGCCAATCCATAAGATAGCCAC). The genomic DNA of strain MGH78578 was used as the template for *mgrB*. The plasmid pDK6 and *mgrB* gene products obtained by PCR were digested by the double digestion method. Then, T4 DNA Ligase (NEB, US) was selected for ligating the pDK6 and *mgrB* gene at 16°C overnight. The pDK6-*mgrB* plasmid was introduced into KQ20786 by electroporation, with pDK6 as control. Transformants were selected on Mueller-Hinton agar containing 30 mg/L apramycin.

### *In vitro* colistin-induced experiment of KQ20605

To ascertain the within-host evolution into colistin resistance of the parental strain KQ20605, the *in vitro*-induced experiment was performed over 6 days, as described in a previous study (Janssen et al., 2020). We first grew the strain KQ20605 in LB broth with initial colistin concentrations of 1 mg/L. After overnight growth, 10-μl cultures were inoculated on MHA plates containing 1, 2, 4, 8, 16, 32, and 64 mg/L colistin and incubated at 37°C for 24 h. At the same time, 15 μl cultures were transferred into a 3-ml fresh LB broth supplemented with the concentration of colistin to double that of the previous day. Well-separated colonies growing on 4 mg/L colistin-containing MHA plates were selected for determining their colistin MICs and identifying the existence and completeness of the *mgrB* genes with PCR (primers: *mgrB*-F 5’-GCCAATCCATAAGATAGCCA-3’ and *mgrB*-R 5’-AGCCAGCGATGCCAGATT-3’) and agarose gel electrophoresis. The PCR products were purified, and Sanger sequencing was performed using ABI 3730 DNA analyzer. The sequences were compared to the database in NCBI Genbank (http://www.ncbi.nlm.nih.gov) using the BLAST algorithm. This micro-evolution of the colistin resistance experiment lasted 6 days.

### Plasmid conjugation experiment

To explore the transferability of the plasmid pKQ20605-IncN co-harboring IS*26* and *bla*_NDM-1_, conjugation experiments were performed using KQ20605 as the donor and *E. coli* strain J53 as the recipient, as previously described (Zhao et al., 2022). The donor and recipient strains were mixed at a ratio of 1:3 and incubated at 37°C for 18 h. Next, the transconjugants were selected on China blue lactose agar supplemented with meropenem (2 mg/L) and sodium azide (100 mg/L). Lastly, the species identification of transconjugants was confirmed by MALDI-TOF MS, and the existence of *bla*_NDM_ was confirmed by the GeneXpert system (Cepheid Inc, Sunnyvale, CA, USA).

### Whole-genome sequencing and bioinformatic analysis

WGS was carried out using Illumina HiSeq 2500 platform and nanopore sequencing method on MinION flow cells for KQ20605, KQ20786, KQ20605-1, KQ20605-2, and KQ20605-5. Raw reads were filtered to remove the low-quality sequences and adaptors using a skewer (Jiang et al., 2014) and PoreChop (https://github.com/rrwick/Porechop), respectively. *De novo* assembly was conducted via SPAdes Genome Assembler v3.13.1 (Prjibelski et al., 2020) and Unicycler (Wick et al., 2017). MLST analysis and identification of antimicrobial resistance determinants and plasmids replicon were confirmed by the Center for Genomic Epidemiology (CGE) (http://genomicepidemiology.org/services/) and Kleborate. The ISfinder database (https://www-is.biotoul.fr) was used to identify insertion sequence elements in the Sanger sequencing data and complete genome sequence. Genome annotation was performed using Prokka v4.13. Genome sequence comparison was performed using the BLAST. CGView was used to visualize the comparison results. Colistin resistance-related genes involving *pmrA*, *pmrB*, *phoP*, *phoQ*, *crrA*, *crrB*, *crrC*, *pmrD*, *pmrK*, *pmrC*, and *mgrB*, were defined by alignment with the *Klebsiella quasipneumoniae* ATCC 700603 genomes through blast+ 2.11.0 with the E value set at 1e-50. Then KQ20605 genome was used as the reference to compare the identity of the above genes among KQ20605, KQ20786, KQ20605-1, KQ20605-2, and KQ20605-5. The PROVEAN platform (http://provean.jcvi.org/index.php) was used to predict alterations in the biological functions of the above-described proteins (Li et al., 2023).

### Phylogenetic analysis

The phylogenetic tree construction was performed to determine the species of the strain KQ20605 and KQ20786. We searched the whole genome sequences with the term “*Klebsiella*” on NCBI, selected the subspecies of *Klebsiella*, including *K. pneumoniae*, *K. variicola*, and *K. quasipneumoniae*, and then re-determined their species by Kleborate 2.0.4 (https://github.com/katholt/Kleborate). The whole genome sequences of 12 clinical isolates worldwide were then downloaded from the GenBank database to build the phylogenetic tree, including 3 K*. pneumoniae* (GCF_000364385.3, GCF_000956385.1, and GCF_001022035.1), 3 K*. quasipneumoniae subsp. quasipneumoniae* (GCF_003285165.1, GCF_020525805.1, and GCF_024917695.1), 3 KQSS (GCF_001060825.1, GCF_001611185.1, and GCF_002248055.2) and 3 K*. variicola* (GCF_000941635.2, GCF_003285185.1, and GCF_003429625.1). For comparative genomic analysis, the KQSS isolate ATCC 700603 (GeneBank: GCA_001596075.2) was selected as the reference genome.

Phylogenetic analysis was performed using Snippy (https://github.com/tseemann/snippy) Gubbins and RaxML. The iTOL (https://itol.embl.de) was used for the visualization of a phylogenetic tree.

### Fitness cost analysis

Briefly, overnight cultures were diluted to an optical density at 600 nm (OD_600_) of ∼0.1 and grown at 37°C with 200 rpm shaking. The culture cell density was determined every 30 min by measuring OD_600_ for 24h. The experiment was repeated three times independently.

### Statistical analyses

GraphPad Prism 8.2.1 was used for data analyses. Significant differences were assessed using a one-way analysis of variance. *P* value <0.05 was considered statistically significant.

## Results

### Case data

**Figure 1:**
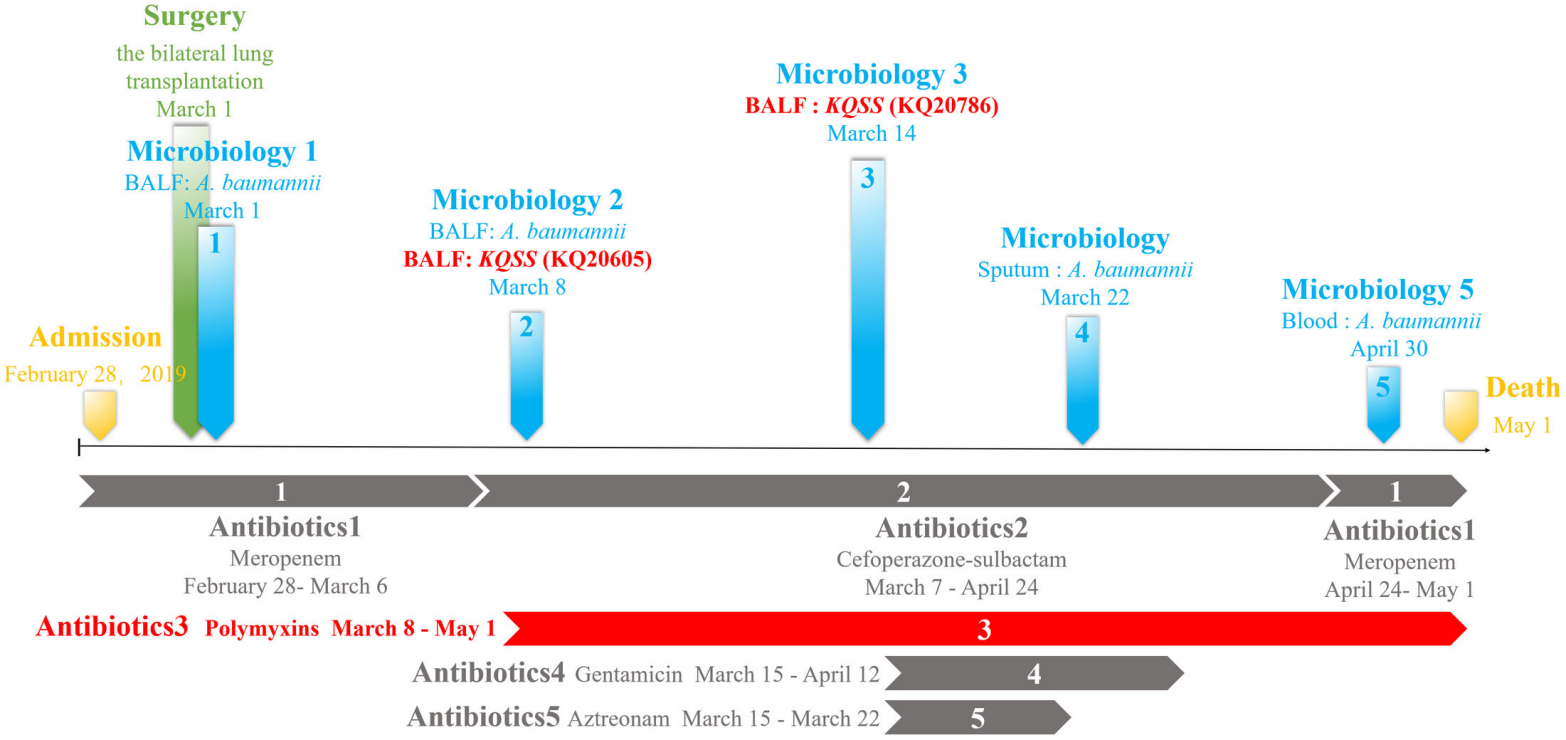
Clinical data of a lung transplantation recipient: time and sites of isolation of KQSS strains and other microbiological results from the patient and antimicrobial treatment. KQSS, *K. quasipneumoniae* subsp. *similipneumoniae*.

In March 2019, a 7-year-old lung-transplanted boy was admitted to National respiratory medicine center, CJFH. The KQSS strain KQ20605, producing metallo-β-lactamase, was isolated from BALF. The strain was susceptible only to colistin, tigecycline, aztreonam, trimethoprim-sulfamethoxazole and aminoglycoside, as shown in **Table 1**. On the 6^th^ day of colistin administration, the patient showed no improvement, and another KQSS strain (KQ20786) was recovered, revealing to be colistin-resistant. Antibiotic treatment with aztreonam was effective, so KQSS was not found. Unfortunately, the patient died soon after due to a bloodstream infection caused by *Acinetobacter baumannii*. The details of the clinical data are shown in **Figure 1**.

**Table 1:** MIC values of different antibiotics to clinical KQSS strains and the induced colistin-induced resistant strains.

Strain ID	source	MIC (mg/L)													
		COL	TIG	IMP	MEM	ATM	TZP	SCF	FEP	CAZ	LEV	AMK	CIP	TOB	MH
KQ20605	BALF	1	2	≥16	≥16	≤1	≥128	≥64	≥64	≥64	1	≤2	2	≤1	4
KQ20786	BLAF	≥64	2	≥16	≥16	≤1	64	≥64	≥64	≥64	2	≤2	2	≤1	4
KQ20786-C	*mgrB*-complemented	0.5	1	≥16	≥16	≤1	≥128	≥64	≥32	≥64	1	≤2	1	≤1	4
KQ20605-1^a^	induced	≥64	2	≥16	≥16	≤1	≥128	≥64	≥32	≥64	4	≤2	2	≤1	8
KQ20605-2^b^	induced	≥64	2	≥16	≥16	≤1	≥128	≥64	≥32	≥64	4	≤2	2	≤1	8
KQ20605-5^c^	induced	≥64	2	≥16	≥16	≤1	≥128	≥64	≥32	≥64	4	≤2	2	≤1	4

KQ20786-C was electroporation transformant of KQ20786 with pDK6-mgrB. KQ20605-1^a^, KQ20605-2^b^ and KQ20605-5^c^ were colistin-induced resistant isolates from parental KQ20605 in vitro. ^a^IS903B element integrated into between the -26^th^ and -25^th^ position of the mgrB gene promoter. ^b^IS26 element integrated into the -20^th^ and -19^th^ position of the mgrB gene promoter. ^c^mgrB-lost. MIC, Minimal inhibitory concentration; KQSS, Klebsiella quasipneumoniae subsp. Similipneumoniae; COL, colistin; TIG, tigecycline; IMP, Imipenem; MEM, Meropenem; ATM, Aztreonam; TZP, Piperacillin-tazobactam sodium; SCF, Cefoperazone-Sulbact; FEP, Cefepime; CAZ, Ceftazidime; LEV, Levofloxacin; AMK, amikacin; CIP, Ciprofloxacin; TOB, Tobramycin; MH, Minocycline.

### Species identification, population analysis profile, and antimicrobial susceptibility tests

**Figure 2:**
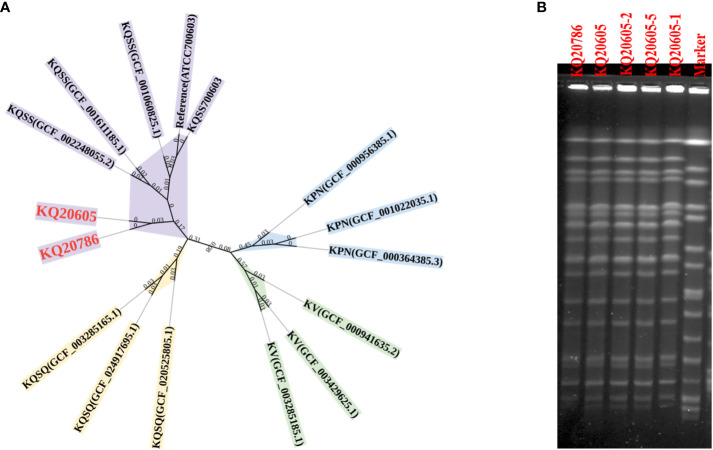
Species and homology analysis on strains in this study. **(A)** Core genome phylogenetic tree. A Core genome phylogenetic tree of 3 KPN (blue), 3 KV (green), 3 KQSS (purple), 3 KQSQ (yellow), KQ20605, and KQ20786 (red bold). ATCC 700603 was as a reference. KPN, *K*. *pneumoniae;* KV, *K. variicola*; KQSS, *K. quasipneumoniae subsp. similipneumoniae*; KQSQ, *K. quasipneumoniae subsp. quasipneumoniae*. **(B)** PFGE of 5 isolates. KQ20605 and KQ20786 were isolated from the patient. KQ20605-1, KQ20605-2, and KQ20605-5 were *mgrB* inactivation-induced isolates *in vitro*. KQ20605-1 (with IS*903B* insertion between -26^th^ and -25^th^ bp in *mgrB*), KQ20605-2 (with IS*26* insertion between -20^th^ and -19^th^ bp in *mgrB*), KQ20605-5 (with a large segment in *mgrB* or *mgrB*-lost). PFGE, Pulsed Field Gel Electrophoresis.

Species identification in the Kleborate database indicated that KQ20605 and KQ20786 belonged to ST2558 and KQSS, initially misidentified as *K. pneumoniae* by MALDI-TOF MS. Pairwise SNP analysis on both strains showed that the SNPs between them was only 21, suggesting that they are clonally related (**Figure 2A**). The genomic identity between KQ20605 and KQ20786 was also confirmed by Pulsed Field Gel Electrophoresis (PFGE), shown in **Figure 2B**. GeneXpert system showed that both isolates carried NDM. The MIC for colistin of KQ20605 was 1 mg/L, and the possibility of colistin-heteroresistance was excluded via PAP. Different from KQ20605, KQ20786 was highly resistant to colistin with a MIC of ≥ 64 mg/L. The AST results are detailed in **Table 1**.

### Comparative genome analysis on KQ20605 and KQ20786 and complementation experiments

**Figure 3:**
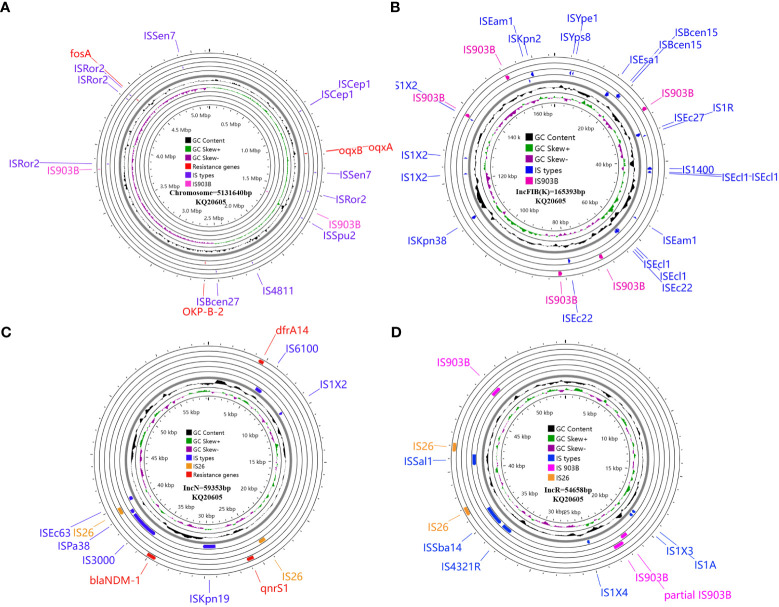
Circular maps and genomic analysis of all insertion sequences distribution and antibiotic resistance genes on the chromosome **(A)**, and plasmids pKQ20605-IncFIB(K) **(B)**, pKQ20605-IncN **(C)**, and pKQ20605-IncR **(D)** of parental KQ20605 KQSS strain. KQSS, *K. quasipneumoniae* subsp. *similipneumoniae*.

**Figure 4:**
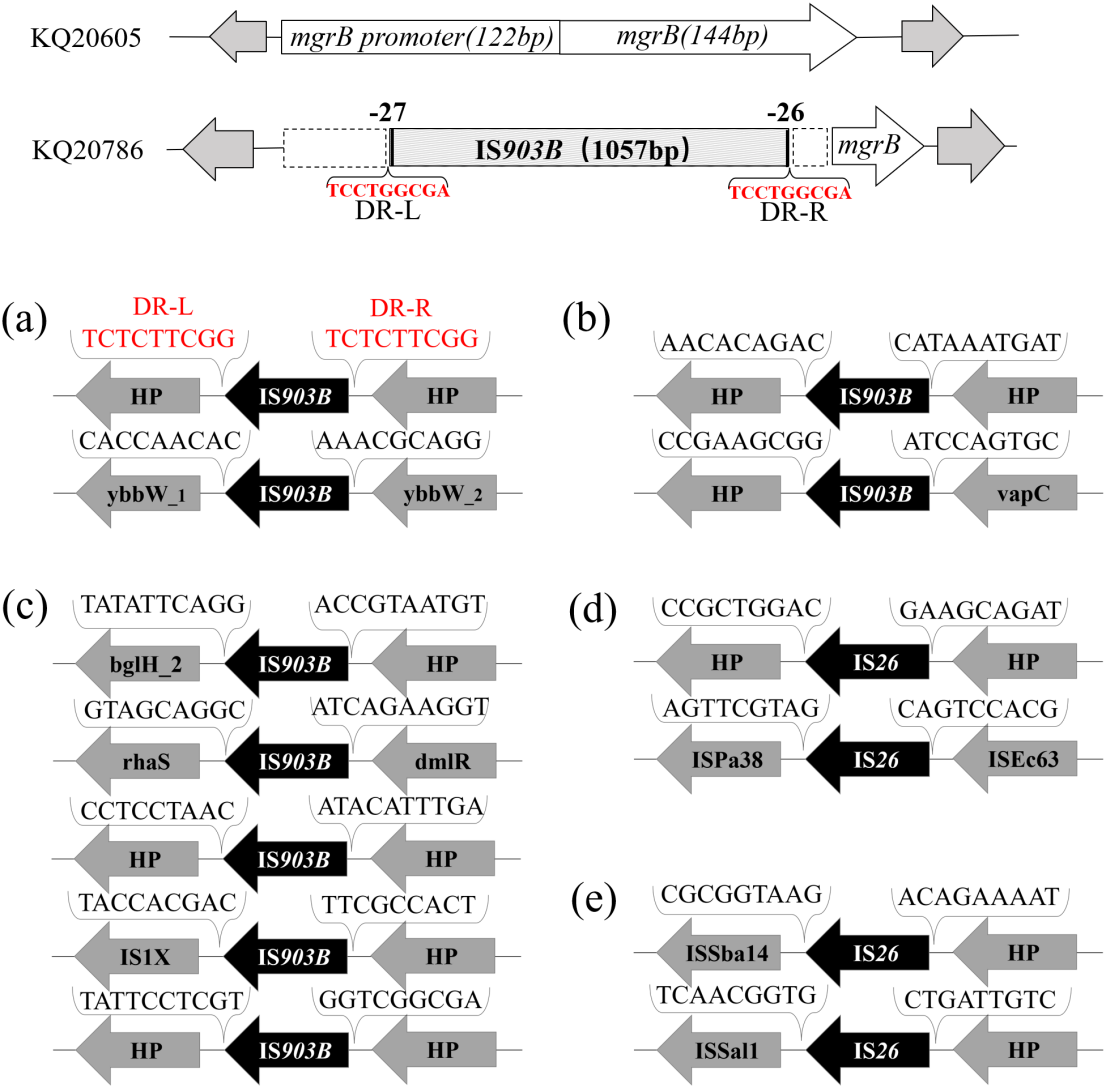
Analysis of insertional inactivated *mgrB* gene of KQ20786 and surrounding genetic environment of △*mgrB*-associated IS elements in KQ20605. **(A)** Visual figure of *mgrB* gene or insertional inactivation *mgrB* gene of KQ20605 and KQ20786. (a) Schematic diagram of *mgrB* genes of the parental KQSS strain (K Q20605) and the colistin-resistant variant (KQ20786) integrated with IS*903B* element between the -27^th^ and -26^th^ bp of *mgrB* promoter. **(B)** Surrounding genetic environment of IS*903B* or IS*26* elements of KQSS strain KQ20605. (a) 2 IS*903B* elements located on chromosome of KQ20605: one flanked by 9-bp direct repeats (DRs) (upper) and the other not (down). (b) 2 IS*903B* elements on plasmid pKQ20605-IncR of KQ20605: neither flanked by DRs, with one (upper) next to a 698-bp incomplete IS*903B* and sharing a 100% (1057/1057) nucleotide identity and coverage match with that on insertional inactivation *mgrB* gene promoter of KQ20786. (c) 5 IS*903B* elements on plasmid pKQ20605-IncFIB(K) of KQ20605: none flanked by DRs. (d, e) 4 IS*26* elements on genome of KQ20605, 2 on plasmid pKQ20605-IncN (d) and 2 on plasmid pKQ20605-IncR (e) respectively: neither flanked by DRs. KQSS, *K quasipneumoniae* subsp. *similipneumoniae*.

The genome of KQ20605 consists of a chromosome (5,131,640 bp) and three plasmids, namely, pKQ20605-IncFIB(K) (165,393 bp), pKQ20605-IncN (59,353 bp), and pKQ20605-IncR (65,658 bp). Both KQ20605 and KQ20786 harbor the resistant genes *fosA, oqxA, oqxB and bla*_OKP-B-2_ on the chromosome, coupled with *qnrS1, dfrA14*, and *bla*_NDM-1_ on pKQ20605-IncN (**Figure 3**). Chromosomal resistance mechanisms for colistin of KQ20786 were determined by investigating the nucleotide sequences and corresponding protein sequences of the genes *pmrA*, *pmrB*, *phoP*, *phoQ*, *crrA*, *crrB*, *crrC*, *pmrD*, *pmrK*, *pmrC*, and *mgrB*, and comparing them to sequences from its parental strain KQ20605. Genome comparison results showed that an IS*903B* was located between the -27^th^ and -26^th^ base pair of *mgrB* promoter in KQ20786 compared to the colistin-susceptible parental strain KQ20605 (**Figure 4A**). The *phoPQ*, *pmrAB*, and *crrAB* genes between KQ20786 and KQ20605 were 100% identical using BLASTn. No *mcr* gene (*mcr*1-10) and its variants were identified in the above two strains. Compensation experiments confirmed that the KQ20786 transformant complemented with pDK6-*mgrB* restored colistin susceptibility (0.5mg/L). The above investigation suggested that continual usage of colistin in the patient resulted in the within-host evolution and selection of colistin resistance through insertional inactivation of *mgrB* gene.

To trace the source of IS*903B* in disruptive *mgrB* of KQ20786, the genomes of KQ20605 were uploaded on ISFinder website and plasmidFinder database, respectively, and the results showed that it carried 9 IS*903B* elements in the genome, including 2 on the chromosome, 5 on pKQ20605-IncFIB(K) and 2 on pKQ20605-IncR, respectively (**Figures 3**, **4B**). Furthermore, to clarify whether the IS*903B* inserted in the chromosomal *mgrB* promoter of KQ20786 was from chromosome or plasmid, this IS*903B* sequence in *mgrB* was aligned with all 9 IS*903B* elements on the whole genome. The comparison result showed that the inserted IS*903B* element in *mgrB* of KQ20786 exhibited 100% (1057/1057) and 99% (1042/1057) identity as well as 100% and 100% coverage to IS*903B* elements on plasmid pKQ20605-IncR of KQ20786 and KQ20605, respectively. By comparison, the identity between IS*903B* inserted into *mgrB* of KQ20786 and each IS*903B* element located on chromosome in KQ20605 or KQ20786 was all only 95.1% (1005/1057), indicating the role of plasmids as possible donors for IS*903B* elements. Also, the upstream of the IS*903B* element that is 100% identical to IS*903B* in *mgrB* gene of KQ20786 was close to a fragmentary IS*903B* (only 698 bp), as shown in **Figure 3D**. In addition, as shown in **Figurs 4B** (a), one of the two IS*903B* elements on the chromosome of KQ20605 was flanked by a 9-bp DRs, but no IS*903B* element on plasmids was flanked by DRs (**Figure 4B** (b) and (c)). Although nearly all IS elements could genetically exchange, the transposition frequency of IS elements with DR at both ends is lower than those without DR (Zhang et al., 2021). Therefore, we suspect that the IS*903B* in mutant *mgrB* gene of KQ20786 is more likely to come from plasmids.

### *In vitro* evolution of colistin resistance of KQ20605

**Figure 5:**
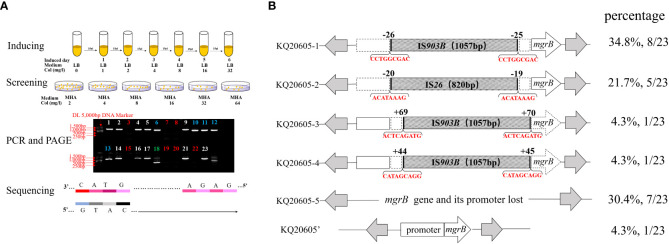
Experimental flow graph and analysis on insertional inactivated *mgrB* gene of *in vitro* colistin-inducted resistant variants **(A)** Schematic diagram of *in vitro* colistin-induced experiment and electropherogram results. a: *In vitro* colistin-induced experiment of KQ20605; b: Well-separated colonies growing on 4 mg/L colistin-containing MHA plates were selected to screen colistin-resistant isolates. c: agarose gel electrophoresis of the colistin-resistant KQSS strains performed on the 3^rd^ day or 6^th^ day of colistin-induced experiments. Strips 1, 2, 4, 5, 9, 14, 16, 17, 21 and 23 indicated IS*903B*-inserted *mgrB* genes, which were highlighted by white color; Strips 6, 10, 11, 12 and 13 were IS*26*-inserted *mgrB* genes, which were highlighted by blue color; Strips 3, 7, 8, 15, 19, 20 and 22 were large segment-inserted *mgrB* genes or *mgrB*-lost, which were highlighted by red color; Strips 18 was intact *mgrB* gene without IS insertion, which was highlighted by green color. d: All confirmed by sequencing. Col, colistin; MHA, Mueller-Hinton agar; AGE, agarose gel electrophoresis. **(B)** Insertional inactivation *mgrB* gene of KQSS strains *in vitro*. Schematic diagram of *mgrB* genes of KQ20605-1 to 5 and KQ20605’, *in vitro* colistin-inducted resistant variants of parental strain KQ20605. *In vitro*, IS*26* or IS*903B* element inserted into *mgrB* gene. All IS*26* elements were inserted between the -20^th^ and -19^th^ bp of *mgrB* promoter (KQ20605-2). IS*903B* elements shared various insertion positions including the -26^th^ and -25^th^, 44^th^ and 45^th^, as well as 69^th^ and 70^th^ bp of *mgrB* gene. The parental strain of these isolates is KQ20605 with an intact *mgrB* gene. Their proportions are listed on the right side of the figure respectively. Each IS element in the disruptive *mgrB* gene was flanked by DRs consisting of 8-9 bp. KQSS, *K. quasipneumoniae* subsp. *similipneumoniae*.

**Figure 6:**
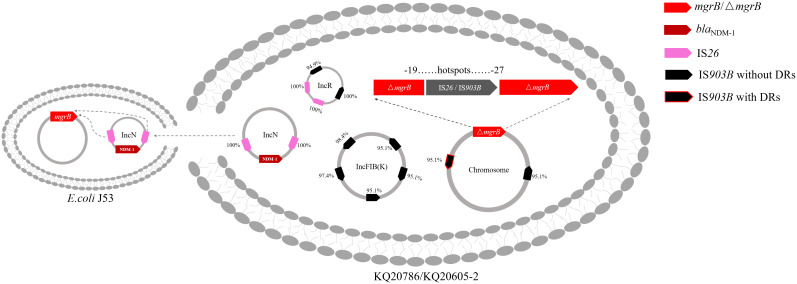
Proposed colistin resistance evolution mechanism of KQ20605 mediated by insertion sequences. The insertion sequence (IS) elements in *mgrB* possibly originated from plasmids. The *mgrB* gene is inserted by IS*26* or IS*903B* at “hotspots” ranging from -27^th^ to -19^th^. Black arrows: IS*903B*; Pink arrows: IS*26*. Arrows with red borders: IS elements flanked by direct repeats (DRs); Bright Red: *mgrB* or mutant *mgrB*; Dark red: *bla*_NDM-1_. Percentage next to IS elements represents the identity between IS on *mgrB* gene and its corresponding IS on genome.

To ascertain if KQ20605 might develop into colistin resistance via *mgrB* gene insertional inactivation and whether the other IS elements on plasmids could also be transferred and inserted into *mgrB* gene under the colistin pressure, the *in vitro* colistin-induced resistance experiment was performed on KQ20605 over 6 days. On the 3^rd^ day, colonies were detected on MHA containing 4mg/L colistin, indicating an *in vitro* transit from a colistin-sensitive to a resistant phenotype occurred under the pressure of colistin. Then, the completeness of *mgrB* genes and promoter regions of 23 randomly selected resistant induced KQSS strains were tested using PCR and Sanger sequencing, and 43.5% (10/23) of them were inserted by IS*903B* elements, 8 between the -26^th^ and -25^th^ bp of *mgrB* promoter (KQ20605-1), 1 between the 69^th^ and 70^th^ bp (KQ20605-3), and 1 between the 44^th^ and 45^th^ bp (KQ20605-4) of *mgrB* gene, as shown in **Figure 5**. In addition, the insertion of another IS type (IS*26* element) into the *mgrB* promoter between the -20^th^ and -19^th^ bp (KQ20605-2) was identified in 21.7% (5/23) colistin-resistant strains. No *mgrB* gene was identified in 30.4% (7/23) strains (KQ20605-5). The BLASTn analysis of WGS for KQ20605-5 indicated that its *mgrB* gene and surroundings (1038bp) were lost. Also, 4.34% (1/23) isolates contained intact *mgrB* gene (KQ20605’). The above strains (KQ20605-1, KQ20605-2, and KQ20605-5) were confirmed to have obvious homology to KQ20605 by AST (**Table 1**), PFGE (**Figure 2B**), species identification as well as morphological characteristics, thus excluding the possibility of contaminations. The *mgrB* inserted by IS elements in all colistin-resistant KQSS strains was flanked by DRs, as shown in **Figures 4B**, **5A**. Apart from IS*903B*, IS*26* elements were also found frequently in inactivated *mgrB* gene in our induced experiment. To decipher their sources, the genome of KQ20605 was uploaded to the ISFinder website and CGE website. There were 4 IS*26* elements, 2 on the pKQ20605-IncN plasmid co-harboring *qnrS1, dfrA14* and *bla*_NDM-1_ and 2 on pKQ20605-IncR, respectively. The IS*26* elements were neither identified on chromosome nor flanked by DRs, as shown in **Figures 3**, **4B** (d) and (e). The 100% identity and 100% coverage between IS*26* inserted in *mgrB* of colistin-induced strains and IS*26* elements on the genome of KQ20605 were identified. Through conjugation experiments, we found that pKQ20605-IncN could transfer to *E.coli* J53 at a frequency of 1.33x10^-6^, thus causing multidrug resistance in the recipient J53. The proposed colistin resistance evolution mechanism of KQ20605 mediated by insertion sequences is summarized in **Figure 6**. Except for *mgrB* and its promoter, no other colistin-resistant mechanisms were detected, such as *pmrA*, *pmrB*, *phoP*, *phoQ*, *crrA*, *crrB*, *crrC*, *pmrD*, *pmrK*, *pmrC*, among KQ20786, KQ20605-1, KQ20605-2 and KQ20605-5 using KQ20605 as reference (**Table S1**).

The growth kinetics of these isolates (KQ20605, KQ20786, KQ20605-1, KQ20605-2, KQ20605-5, and KQ20786-C) were independently investigated 3 times to determine whether the different IS types in insertional inactivation *mgrB* brought different fitness costs on KQSS strains. As shown in **Figure S1**, no significant difference was detected in the growth rates of *mgrB* inactivation-induced isolates and KQ20786 in LB compared to its parental isolate KQ20605. Also, no impact was detected on bacterial fitness from the complementation.

## Discussion

The prevalence of carbapenem-resistant *Klebsiella* spp. has been a major public health concern worldwide (Biswas et al., 2012; Grundmann et al., 2017; Binsker et al., 2022). Colistin is considered the “last resort” treatment for carbapenem-resistant *Klebsiella* spp. In this study, we reported a pair of genetically sequential and NDM-producing *Klebsiella* strains from a lung transplant child and explored the underlying mechanism of transition from a colistin-susceptible strain (KQ20605) to the resistant one (KQ20786) after 6-day colistin exposure.

Previous studies have suggested that the chromosomal *mgrB* gene might be disrupted by IS elements integration and result in colistin resistance (Cannatelli et al., 2014; Olaitan et al., 2014; Poirel et al., 2015; Esposito et al., 2018; Shankar et al., 2019; Yang et al., 2020). For example, of 32 colistin-resistant *K. pneumoniae* isolates from Taiwan, 21 had their *mgrB* genes inserted by IS elements and 1/21 by IS*903B*-like at +44^th^ bp of *mgrB* (Yang et al., 2020). Similarly, Olaitan et al. studied *mgrB* insertional inactivation in 6 of 12 *mgrB*-disruptive and colistin-resistant clinical isolates collected in Lao PDR, Thailand, Israel, Nigeria, and France, with 3 isolates having IS*903B*-like insertion at nt95, nt82 and nt26 of *mgrB*, respectively (Olaitan et al., 2014). Several authors have attributed the rise and spread of colistin resistance in *Klebsiella* to insertional inactivation of the *mgrB* gene by IS elements from plasmids (Giordano et al., 2018; Shankar et al., 2019; Zhang et al., 2021; Fordham et al., 2022). However, clinical data and *in vitro* experiments that could confirm this hypothesis are lacking.

In the current study, the IS*903B* element in KQ20605 was mobilized and integrated into the *mgrB* gene promoter, resulting in colistin resistance (KQ20786) after colistin administration. Comparative genomic analysis of both strains revealed that the plasmid pKQ20605-IncR might serve as a donor for IS*903B* elements integrated into the chromosomal *mgrB* gene. The IS*903B* transposed into chromosomal *mgrB* gene has a 100% (1057/1057) nucleotide identity and coverage match with one IS*903B* element on plasmid pKQ20605-IncR, and only 95.1% (1005/1057) nucleotide identity to that on the chromosome. In addition, 9 IS*903B* elements on the genome, 2 on the chromosome, and 7 on plasmids (pKQ20605-IncFIB(K) and pKQ20605-IncR), were found. It was found that IS*903B* elements tended to be distributed on plasmids to mediate colistin resistance, especially on the fusion plasmid IncHI2A + IncHI2, IncFIB(K) and IncR (Fordham et al., 2022). Also, one IS*903B* on the chromosome was flanked by DRs, whereas none of the IS*903B* elements on plasmids were flanked. Previous studies confirmed that the IS element (IS*Kpn72*) with DRs had a lower transposition frequency than without (Zhang et al., 2021). To sum up, it is reasonable to hypothesize that IS*903B* elements on plasmids rather than on chromosomes were more inclined to be integrated into the chromosomal *mgrB* gene, thereby causing colistin resistance. Thus, further experiments have been performed to prove our hypothesis.

In the *in vitro* colistin-induced experiment of the KQ20605 strain, the mutation types of the *mgrB* gene varied, involving insertional inactivation at “hotspots” sites of the *mgrB* gene and the loss of it, which revealed that the inactivation of the *mgrB* gene is an inevitable event in this strain under the colistin pressure. Specifically, 65.2% (15/23) of colistin-induced isolates were inserted by various IS elements (e.g., IS*903B* and IS*26*) in the *mgrB* gene. Among these 15 isolates, 8 strains were integrated IS*903B* element between the -26^th^ and -25^th^ bp of the *mgrB* gene, causing colistin resistance, an almost identical “hotspot” found in our patient. Moreover, 5 colistin-induced resistant isolates (KQ20605-2) were identified with the integration of IS*26* element between the -20^th^ and -19^th^ bp of the *mgrB* gene. The inserted sites in the *mgrB* gene of this isolate were frequently focused on promoter regions ranging from -27^th^ to -19^th^ bp. Taken together, our data suggest the existence of insertional preferred sites, namely, “hotspots”, in the *mgrB* gene, both *in vivo* and *in vitro* under specific conditions, as also documented in other insertional inactivated *mgrB* mutants (Fordham et al., 2022).

The IS*26* element, commonly located on antibiotic-resistant plasmids (Partridge et al., 2018) has been rarely reported to insert *into* the *mgrB* gene (Silva et al., 2021). A previous study confirmed that the IS*Kpn72*-and-*bla*_KPC_ harboring mobile plasmid enables the strain to develop colistin heteroresistance from susceptibility by integrating IS*Kpn72* into the *mgrB* gene from the mobile plasmid (Zhang et al., 2021). However, proof of the role of IS*26* elements in the emergence and dissemination of colistin resistance was lacking. Comparative genomic analysis showed that the IS*26* elements were all located on plasmids (pKQ20605-IncR and pKQ20605-IncN), and none of them were flanked by DRs in KQ20605, further demonstrating that plasmids served as donors to encode mobilizable IS elements integrating into the chromosomal *mgrB* gene. Moreover, the plasmid pKQ20605-IncN of the KQ20605 strain, co-harboring *qnrS1, dfrA14, and bla*_NDM-1_, could be conjugated into *E. coli* J53, hinting that under the colistin pressure, the IS*26* elements on the carbapenem-resistant plasmid pKQ20605-IncN could be activated and mobilized into chromosomal *mgrB* gene, thereby causing the development, and even dissemination of colistin resistance.

In conclusion, we identified a colistin-resistant KQSS strain transited from its sensitive parental strain by acquiring an IS element in the chromosomal *mgrB* gene promoter in the process of using colistin. Furthermore, we provided clinical data and experimental evidence for the possibility that plasmids preferred to encode mobilizable IS elements, resulting in the insertional inactivation of chromosomal *mgrB* gene at “hotspots”, and subsequently causing the colistin resistance emergence and further potential dissemination.

## Data availability statement

The datasets presented in this study can be found in online repositories. The names of the repository/repositories and accession number(s) can be found below: https://www.ncbi.nlm.nih.gov/, BioProject: PRJNA985803.

## Ethics statement

Permission for using the information in the medical records of the patient and the KQSS isolates for research purposes was granted by the Ethics Committee of the China-Japan Friendship Hospital (CJFH) (2022-KY-054).

## Author contributions

YW, JZ, ZL, XL, FZ, YH, DP, XZ, HS, YZ, CL and BL collected the clinical and laboratory data. YW and BL made substantial contributions to conception and design, drafted, reviewed, and edited the manuscript. All authors contributed to the article and approved the submitted version.
