# Supplementary material for: Within-host acquisition of colistin-resistance of an NDM-producing Klebsiella quasipneumoniae subsp. similipneumoniae strain through the insertion sequence-903B-mediated inactivation of mgrB gene in a lung transplant child in China
Source: Front Cell Infect Microbiol. 2023 Aug 31;13:1153387. doi: 10.3389/fcimb.2023.1153387 (PMC10513040; doi:10.3389/fcimb.2023.1153387)
Supplement: Supplementary file 1 [file DataSheet_1.docx]

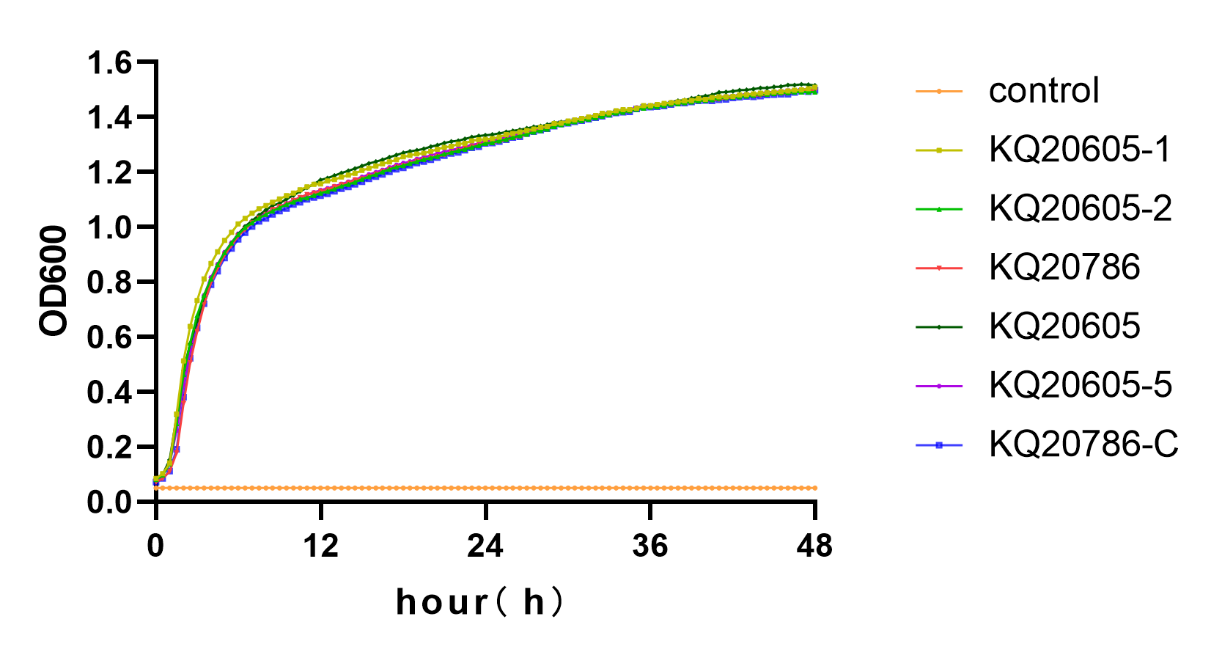


Figure S1. Growth kinetics in LB broth. Data are presented as the mean ± standard deviation of three independent experiments.
