# Supplementary material for: Within-host acquisition of colistin-resistance of an NDM-producing Klebsiella quasipneumoniae subsp. similipneumoniae strain through the insertion sequence-903B-mediated inactivation of mgrB gene in a lung transplant child in China
Source: Front Cell Infect Microbiol. 2023 Aug 31;13:1153387. doi: 10.3389/fcimb.2023.1153387 (PMC10513040; doi:10.3389/fcimb.2023.1153387)
Supplement: Supplementary file 2 [file Table_1.docx]

| **Strains** | **Colistin resistance-related genes** | | | | | | | | | | |
| --- | --- | --- | --- | --- | --- | --- | --- | --- | --- | --- | --- |
|  | ***mgrB* and promoter** | ***phoP*** | ***phoQ*** | ***pmrA*** | ***pmrB*** | ***crrA*** | ***crrB*** | ***crrC*** | ***pmrD*** | ***pmrC*** | ***pmrK*** |
| KQ20786 | IS*903B* insertion in -27bp | 100% | 100% | 100% | 100% | 100% | 100% | 100% | Not detected | 100% | 100% |
| KQ20605-1 | IS*903B* insertion in -26bp | 100% | 100% | 100% | 100% | 100% | 100% | 100% | Not detected | 100% | 100% |
| KQ20605-2 | IS*26* insertion in -20bp | 100% | 100% | 100% | 100% | 100% | 100% | 100% | Not detected | 100% | 100% |
| KQ20605-5 | Deletion (1038bp) | 100% | 100% | 100% | 100% | 100% | 100% | 100% | Not detected | 100% | 100% |

**Table S1 Colistin resistant mechanisms of KQ20786 and *in vitro* colistin-induced resistance strains**

Notes: KQ20786 was isolated in *vivo* and KQ20605-1, KQ20605-2 and KQ20605-5 were colistin-induced resistant isolates from parental KQ20605 *in vitro*. The KQ20605 was used as reference.
